# Supplementary material for: Influence of Block Ratio on Thermal, Optical, and Photovoltaic Properties of Poly(3-hexylthiophene)-b-poly(3-butylthiophene)-b-poly(3-octylthiophene)
Source: Molecules. 2022 Dec 2;27(23):8469. doi: 10.3390/molecules27238469 (PMC9736563; doi:10.3390/molecules27238469)
Supplement: Supplementary file 1 [file molecules-27-08469-s001.zip › molecules-2062760-supplementary.pdf]

Supplementary Materials

Influence of Block Ratio on Thermal, Optical, and Photovoltaic Properties of poly(3-hexylthiophene)-*b*-poly(3-butylthiophene)-*b*-poly(3-octylthiophene)

Van Hai Nguyen[1], Thanh Danh Nguyen[1,2], Jongwoo Song[1], Jongdeok An[1], and Chan Im[1,*]

[1]*Department of Chemistry, Konkuk University, 120 Neungdong-ro, Gwangjin-gu, Seoul 05029, South Korea*

[2]*Institute of Chemical Technology, Vietnam Academy of Science and Technology, 1A, TL29, Thanh Loc Ward, District 12, Ho Chi Minh City 700000, Vietnam*

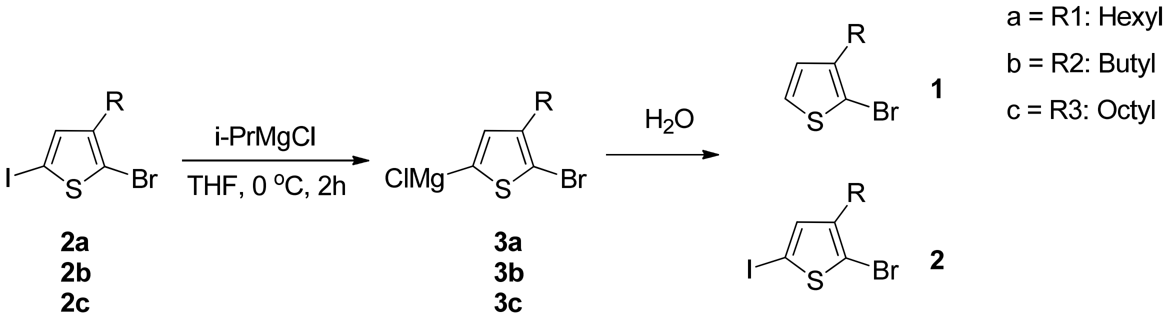

Scheme S1. Procedure to verify the degree of monomer conversion using the Grignard reagent.

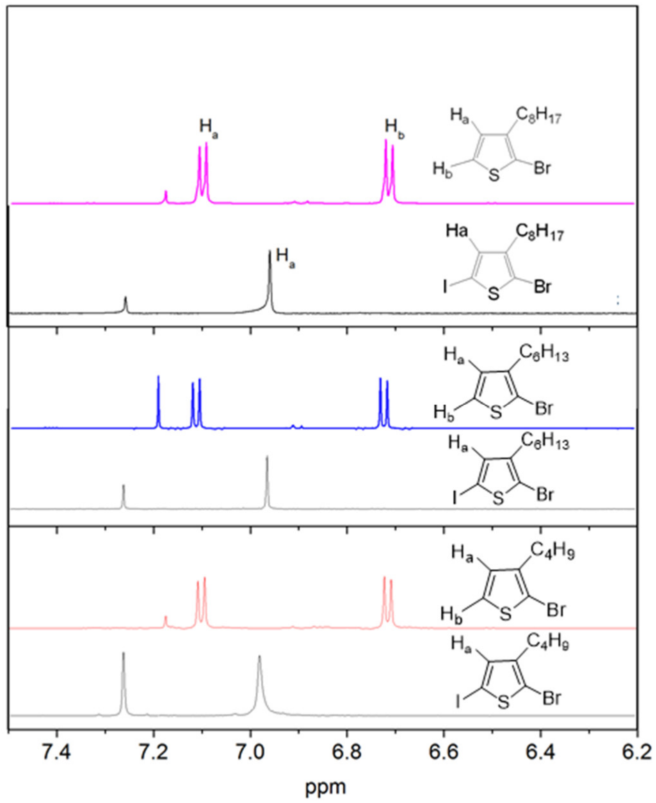

Figure S1. ^{1}H-NMR spectra of monomers for P3OT, P3HT, and P3BT before and after quenching to verify the degree of monomer conversion with the Grignard reagent.

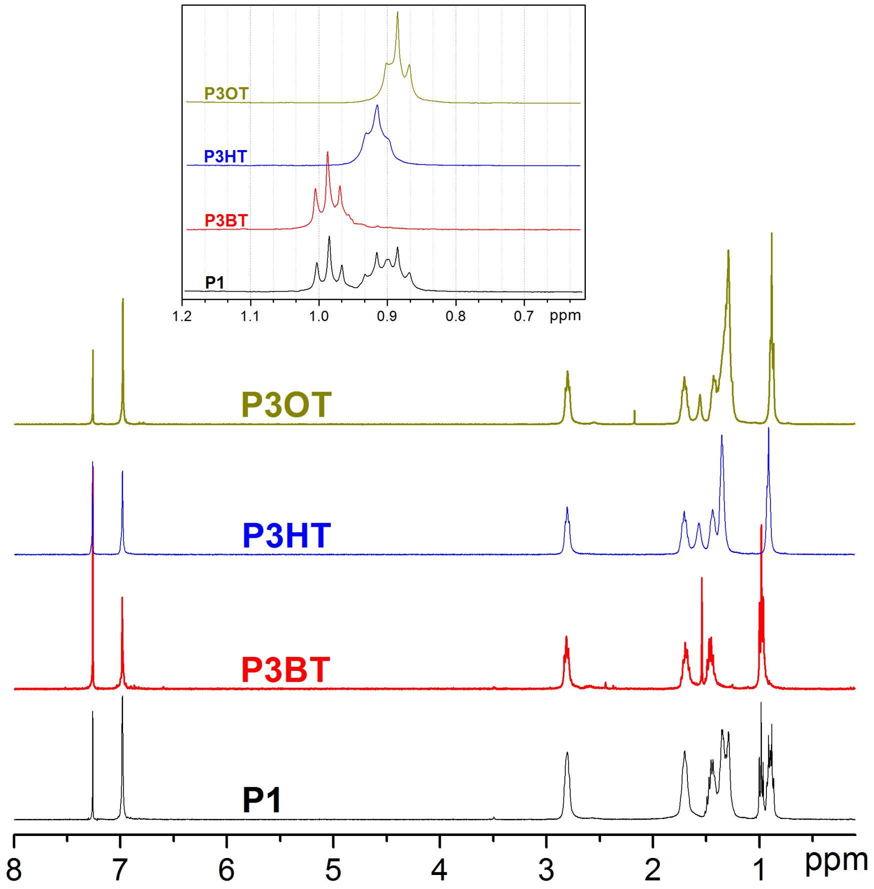

Figure S2. ^{1}H-NMR of homopolymers and copolymer P1.

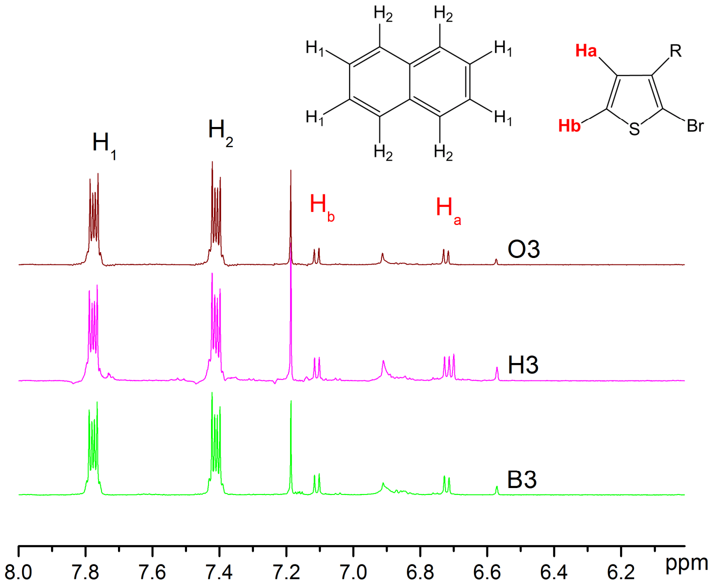

Figure S3. ^{1}H-NMR spectra of the three types of monomers with naphthalene as internal standard.

Table S1. Parameters for calculation of yield using relative qNMR method.

**Compounds**	Parameters	H_a (monomer)	H_b (monomer)	H_1 (naphthalene)	H_2 (naphthalene)
**B3**	I	0.025	0.024	1.0	1.0
	N	1	1	4	4
	n_x/n_y (mean)	0.1			
	Yield	90%			
**H3**	I	0.023	0.021	1.0	1.0
	N	1	1	4	4
	n_x/n_y (mean)	0.088			
	Yield	91%			
**O3**	I	0.017	0.018	1.0	1.0
	N	1	1	4	4
	n_x/n_y (mean)	0.070			
	Yield	93%			

$$\frac{n_x}{n_y} = \frac{I_x}{I_y}\frac{N_y}{N_x}$$ (Used equation, J. Pharm. Biomed. Anal. 38 (5), 2005, 813)

n_x/n_y: mol ratio between monomer and naphthalene.

I_x, I_y: integrated signal areas of monomer and naphthalene.

N_x, N_y: proton number of monomer and naphthalene.

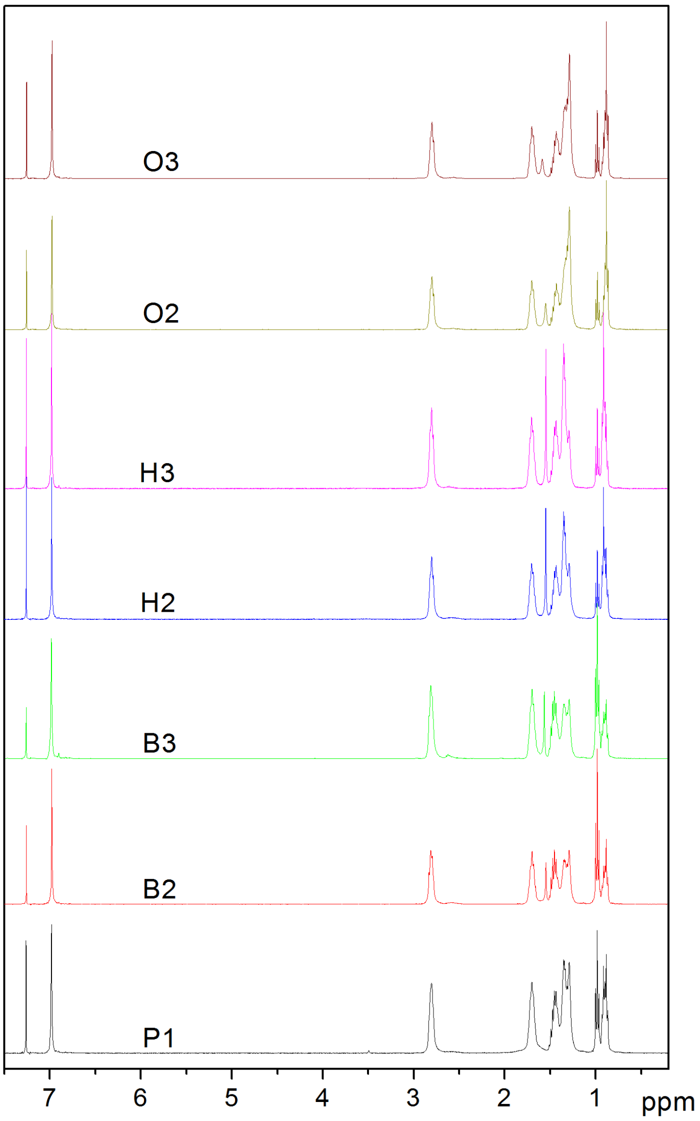

Figure S4. ^{1}H-NMR spectra of the synthesized triblock copolymers.

Table S2. Calculated IQE at 500 nm of block copolymer devices.

Block copolymer	IPCE /%	Reflectance /%	IQE /%
**P1**	54.6	14.8	77.2
**H2**	58.2	12.3	80.0
**H3**	61.8	12.3	85.0
**B2**	56.3	12.6	77.7
**B3**	56.9	12.3	78.3
**O2**	52.6	14.9	76.3
**O3**	55.8	13.6	77.9

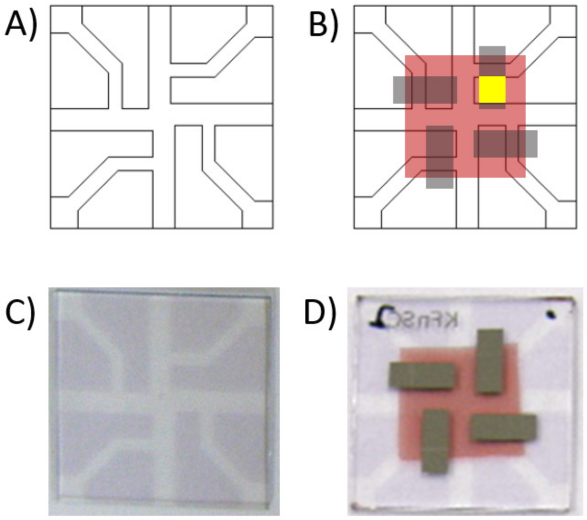

Figure S5. A), B) Structural schema and C), D) actual photos of a studied OPV devices. A) and C) are patterned ITO covered glass substrates. Blueish areas in C) and D) are ITO covered parts. The red rectangles in B) and D) are active layers intercalated between a bottom ITO electrode and a top metal electrode. The overlap areas between ITO and metal electrodes are active areas with 3 mm × 3 mm dimension. One pixel is marked with yellow color for better overview. The active layer shown in D) is a BHJ type active layer consists of P3HT:PCBM blend film.

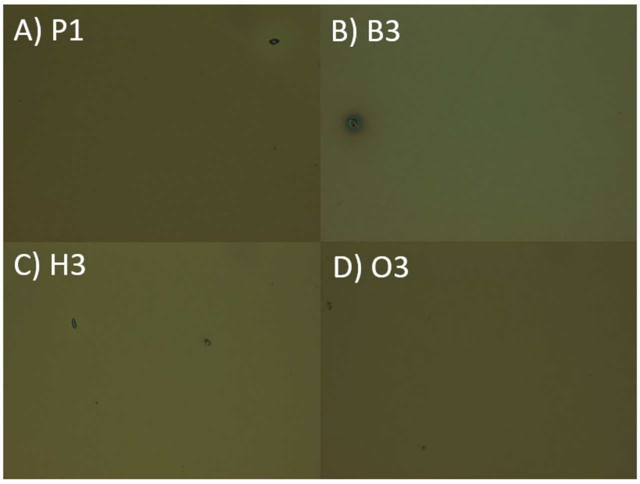

Figure S6. Conventional microscopic images of selected triblock copolymer pristine films after annealing. Each image has a dimension of 100 μm × 120 μm.
